# Divergence in the Morphology and Energy Metabolism of Adult Polyphenism in the Cowpea Beetle *Callosobruchus maculatus*

**DOI:** 10.3390/insects16010029

**Published:** 2024-12-30

**Authors:** Zhong Du, Xiaokun Liu, Sipei Liu, Lei Jiang, Le Zong, Wenjie Li, Weili Fan, Lijie Zhang, Fengming Wu, Siqin Ge

**Affiliations:** 1 Key Laboratory of Zoological Systematics and Evolution, Institute of Zoology, Chinese Academy of Sciences, Beijing 100101, China; duzhong23@ioz.ac.cn (Z.D.); liuxiaokun22@ioz.ac.cn (X.L.); spliu@ioz.ac.cn (S.L.); bugarmy@foxmail.com (L.J.); zongle@ioz.ac.cn (L.Z.); liwenjie@ioz.ac.cn (W.L.); 15110637937@163.com (W.F.); 2 University of Chinese Academy of Sciences, Beijing 100101, China; 3 Science and Technical Research Center of China Customs, Beijing 100101, China; zhanglijie8820@163.com

**Keywords:** *Callosobruchus maculatus*, dimorphism, morphology, transcriptome, fertility

## Abstract

Adult polyphenism is a prevalent form of adaptive evolution that enables insects to generate discrete phenotypes based on environmental factors. However, the morphology and molecular mechanisms underlying adult dimorphism in *Callosobruchus maculatus* (a global storage pest) remain elusive. Understanding these mechanisms is crucial for predicting the dispersal and population dynamics of *C. maculatus*. This knowledge can also provide a theoretical basis for biological control strategies. In this study, we compared the morphology of the hind wing and chest muscles, the transcriptional profiles, the energy metabolism substances, and the fecundity between the flight form and the normal form. The flight form displays a lighter overall appearance with small black spots, while the normal form lacks most flight muscles. Moreover, there are differences in the energy metabolism pathways between the two forms, including carbohydrate metabolism and oxidative phosphorylation. The flight form exhibits higher contents of carbohydrates, lipids, and mitochondrial energetic storage. The normal form exhibits better fertility but has lost its ability to fly. This is the first study to analyze the morphology and molecular characteristics of adult polyphenism in *C. maculatus* using morphological, physiological, and behavioral approaches, providing a foundational understanding of these aspects. Our study on *C. maculatus* also provides supporting evidence of a trade-off between dispersion and reproduction, where the flight form is capable of flying while the normal form has more reproductive benefits.

## Simple Summary

Adult polyphenism is a prevalent form of adaptive evolution that enables insects to generate discrete phenotypes based on environmental factors. *Callosobruchus maculatus*, belonging to Coleoptera, Chrysomelidae, Bruchinae, is a global storage pest known for causing widespread harm. However, the morphology and molecular mechanisms underlying adult dimorphism in *C. maculatus* remain elusive. Our results reveal that the enhanced development of the flight muscles and robust energy metabolism in the flight form facilitate their ability to fly, based on their morphological, physiological, and behavioral traits, but those come at the cost of abnormal development of their reproductive organs. The results of this study on *C. maculatus* also support evidence of a trade-off between dispersion and reproduction. Understanding the morphology and molecular mechanisms underlying adult dimorphism in *C. maculatus* is crucial for predicting its dispersal and population dynamics. This knowledge can also provide a theoretical basis for biological control strategies.

## 1. Introduction

Polyphenism refers to the phenomenon where the same genotype or genome gives rise to two or more different phenotypes, with individual differences being induced by various environmental factors. It is a prevalent form of adaptive evolution that enables insects to generate discrete phenotypes. It can also be categorized into seasonal or dispersal polyphenism depending on external factors. In the study of seasonal polyphenism, the wing patterns of butterflies and the reproductive patterns of aphids, for example, are influenced by seasonal changes. Gregarious and solitary locusts and winged and wingless aphids are associated with dispersal polyphenism. Gregarious locusts can fly for longer distances at relatively lower speeds, while solitary locusts possess high initial flight speeds and engage in short-term flights [1,2,3]. Pea aphids can produce genetically identical winged and wingless offspring in response to environmental factors such as temperature and population density [4,5]. Winged aphids rely on flight ability to colonize new habitats and avoid competition for resources, but this comes at the cost of reduced reproduction [6,7]. *Velarifictorus aspersus* (Walker) is a cricket species widely distributed throughout China that displays distinct wing dimorphism [8,9]. Due to differences in temperature exposure and density conditions, the crickets can be classified as either long-winged or short-winged. Long-winged individuals are capable of flight, while short-winged individuals have more reproductive benefits. This suggests a trade-off between dispersion and reproduction in crickets [10,11].

*Callosobruchus maculatus* belongs to Coleoptera, Bruchinae. It is a global storage pest that causes widespread harm. Adult polymorphism in *C. maculatus* was first reported by Utida, who designated the form with flight ability was named “flight form” [12]. Studies have found that temperature, seed water content, and larval density contribute to the emergence of a high number of flight forms [13,14]. The flight form is mainly active in the field, while the normal form primarily resides in warehouse environments [15], indicating that flight may play a crucial role in the dispersion of *C. maculatus*. In recent years, studies have mainly focused on the oviposition preference, life history, population growth, and photopreference of *C. maculatus* [16,17,18,19,20]. However, the morphology and molecular mechanisms underlying adult dimorphism remain elusive. Understanding these mechanisms is crucial for predicting the dispersal and population dynamics of *C. maculatus* and can also provide a theoretical basis for biological control strategies.

In this study, we investigate the mechanisms of adult polyphenism in *C. maculatus* using a combination of morphological, physiological, and behavioral approaches. This is the first study to analyze the morphology and molecular characteristics of adult polyphenism in *C. maculatus*, providing a foundational understanding of these aspects. Our studies on *C. maculatus* also provide supporting evidence of a trade-off between dispersion and reproduction.

## 2. Materials and Methods

### 2.1. Insect Rearing

*C. maculatus* beetles were obtained from a shipment of cowpeas from Nigeria, intercepted by Beijing Customs. In this study, the beetles were maintained and reared on a standard medium of cowpea seeds under controlled conditions at 25 ± 1 °C, 60% ± 10% RH, and 12:12 h LD at the Institute of Zoology, Chinese Academy of Sciences, Beijing, China.

### 2.2. Wing Measurement

The adults were cold-anesthetized, and their hind wings were dissected and flattened under a stereo microscope. Photographs of the hind wings were taken using a Leica EM CPD 300/SCD050. The length of the hind wings was measured from the humeral angle to the apical angle, while the wing width was measured from the intersection of the costal and subcostal veins to the Cu2 terminal.

### 2.3. Three-Dimensional (3D) Reconstruction

The samples were subjected to micro-CT scans using beam settings of 60 kV and 133 µA. The resulting micro-CT image stack was used to reconstruct a three-dimensional structure of the thorax using Amira 6.1.1 (Thermo Fisher Scientific Bartlesville, America). VG Studio Max (Volume Graphics Heidelberg, Germany) 3.0 was used for volume rendering, and Adobe Illustrator CC 2018 software, version 22.1 was used for typesetting.

### 2.4. Transcriptome Sequencing

Total RNA was extracted from ten unmated random samples of both the flying form and the normal form using TRIzol reagent (Ambion Austin, America #15596018), and each treatment was repeated three times. cDNA library construction was performed using the NEB Next Ultra RNA Library Prep Kit, and Illumina sequencing of the samples was conducted on the Illumina novaseq6000 platform at Novogene Co., Ltd., in Beijing, China. The raw data (SR27078116 and SRX27078116) were filtered, and then the cleaned data were mapped to the C. maculatus genome sequence using the HISAT2 package [21]. The DEseq2 package was used to detect differentially expressed genes (DEGs), and the pheatmap package was used for analysis of the fragments per kilobase of transcript per million mapped reads (FPKM), generating outputs in the form of a heat map.

### 2.5. Quantitative Real-Time PCR (qPCR)

Total RNA from ten unmated random samples of the two forms was prepared using TRIzol reagent (Ambion #15596018), and each treatment was repeated three times. The cDNA was synthesized using the Prime Script reagent kit (Takara Kyoto, Japan #RR047A). qPCR was performed on the Thermo Piko Real 96 (Thermo) using the SYBR Green PCR Master Mix (Takara #RR820A). The transcription levels of the genes were calculated using the 2^–ΔΔCt^ method [22], with *BTF3* used as the housekeeping gene. The primers used in this study are as follows (Table 1):

### 2.6. Energy Metabolism Substance Measurement

Ten random samples of unmated insects (*C. maculatus*) were homogenized in 1 mL of PBST (0.1% Tween 20 in PBS) and immediately incubated at 70 °C for 5 min, then centrifuged at 4 °C at 12,000× *g* for 5 min. Next, we took 200 µL of the supernatant and diluted it 5 times. The diluted solution was used for the subsequent energy metabolism substance experiment [23,24], and each treatment was repeated three times. The protein concentration in the homogenate was determined using a chemical kit from Solarbio (BC3180). The contents of the energy metabolism substances, including glycogen, D-trehalose, triglyceride, acetyl-CoA, NAD, and NADH, were measured using chemical kits from Solarbio (BC0340, BC0330, BC0620, BC0980, and BC0310) (Beijing, China) according to the manufacturer’s protocols. The measured values were normalized based on the protein levels in each homogenate.

### 2.7. External Morphology of Reproductive Organs

A female was cold-anesthetized, and the reproductive organ was dissected using a stereo microscope. Then, the reproductive organ was mounted on slides in a drop of glycerol for photographing. The images were captured using a Canon EOS R5 digital camera (with a 5× objective lens), and all the images were stacked using Helicon Focus 7.7.4.

### 2.8. Egg-Laying Assay

To evaluate the egg-laying of both forms of mated females, virgin females were first mated with males in a transparent plastic Petri dish (90 mm). Subsequently, the 3 mated females were transferred to a new transparent plastic Petri dish containing cowpea seeds. The numbers of eggs laid in the cowpea seeds were manually counted under a stereo microscope after 3 days.

### 2.9. Statistical Analysis

The wing length, wing width, and energy metabolism substance measurement data were analyzed using an unpaired *t*-test. The egg position data were analyzed using one-way ANOVA and post hoc Tukey’s test. All statistical analyses were conducted using Prism7 (GraphPad software) version 7.05.

## 3. Results

### 3.1. External Morphology of Flight Form and Normal Form

**Figure 1:**
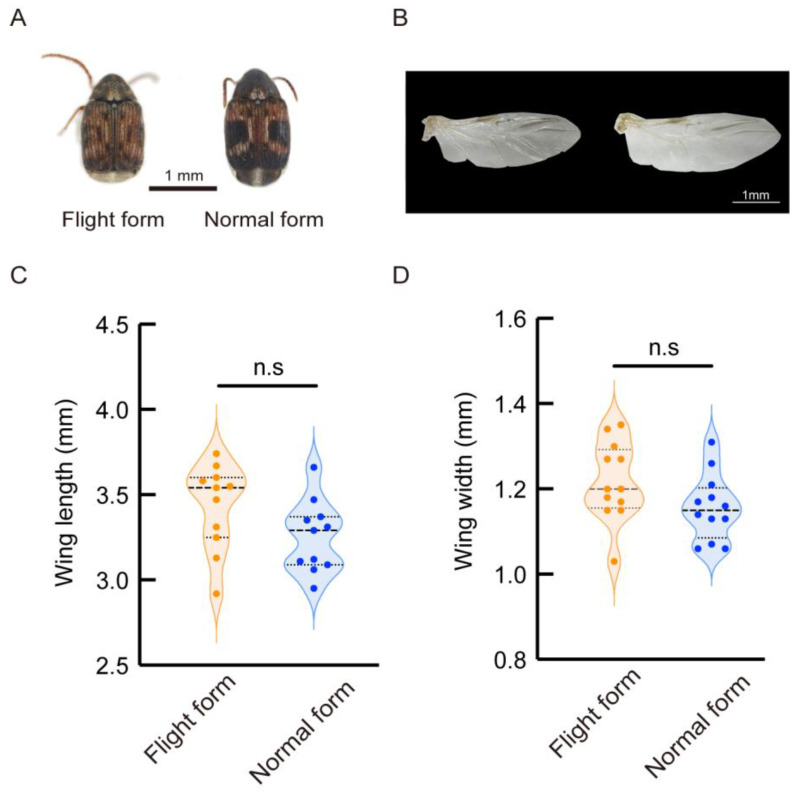
The external trait between flight form and normal form adult. (**A**) The external morphology in flight and normal form adult; (**B**) the external morphology of the wing in flight and normal form; (**C**) wing length; (**D**) wing width. n.s indicates no significant difference (unpaired *t*-test).

Based on our observations, we found that the flight form displayed a lighter overall appearance. The normal form had a darker overall coloration. Both forms of females had black spots on their shoulders, centers, and tips of the elytra, with the largest spot located in the center. In the normal form, the central black spot on the elytra was larger and darker compared to that of the flight form [Figure 1A]. The wings of both forms generally had a similar appearance, except that the anal area of the normal form was slightly larger than that of the flight form [Figure 1B]. The statistical results showed no difference in the wing length or width between the two forms [Figure 1C,D].

### 3.2. Internal Muscles of Flight Form and Normal Form

**Figure 2:**
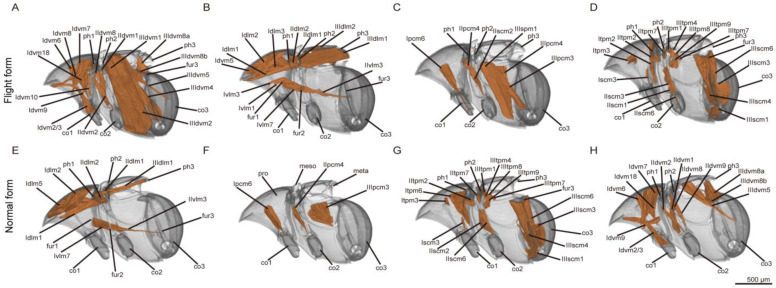
Three-dimensional reconstructions of the thoracic muscles of flight form and normal form. (**A**–**D**) Thoracic muscles of flight form: (**A**–**D**) are the profile view of the thorax of flight form, but show different layers of thoracic muscles. (**E**–**H**) Thoracic muscles of normal form: (**E**–**H**) are the profile view of the thorax of normal form, but show different layers of thoracic muscles. Muscle nomenclature and abbreviations are used following Friedrich and Beutel (2008) [25].

To evaluate potential differences in the internal muscles between the two forms, we examined their internal muscular structures via a three-dimensional (3D) reconstruction technique. The musculature system of the thorax, in both the flight and normal forms, is described and illustrated in Figure 2. The large indirect flight muscle system of the flight form primarily consists of the dorsal longitudinal muscles (dlm) and the dorsoventral muscles (dvm): IIdlm1 and 2, IIIdlm1 and 2, IIdvm1, 2, 8 and 9, IIIdvm1, 2, 5, 6 and 8. These muscles occupy the majority of the muscular mass in the pterothorax and generate the main force required to deform the thorax, providing the power for flapping. The direct flight muscles, which are attached to the basalare and axillary sclerites are generally small, including the tergo-pleural muscles (tpm): IItpm2 and 7, IIItpm1, 4, and 7–9. These muscles influenced wing movements by controlling the joints at the wing base. Some of these muscles, such as IIIpcm2–4, might function as both indirect and direct flight muscles by connecting to the basalare. It should be noted that beetles rely on the muscles in the metathorax (III) for fighting, while the muscles in the mesothorax (II) are responsible for opening and closing the elytra. The normal form lacks muscles IIspm7, IIIdlm2, and IIIdvm1, 2 and 6 compared to the flight form, but it possesses muscle IIdvm9. In addition, the normal form also lack prothoracic muscles Idlm3, Idvm8 and 10, and Ivlm1 and 3. The prothoracic musculatures differ between the two forms of beetles.

### 3.3. Identification of Differential Gene Expression

**Figure 3:**
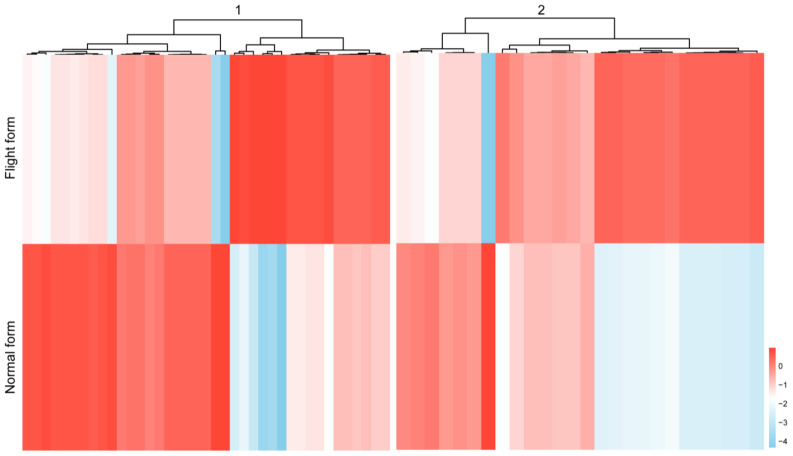
The expression heatmap of energy metabolism gene between flight form and normal form. Expression patterns of gene clusters involved in carbohydrate metabolism (1) and oxidative metabolism (2). In the heatmap, the color scale represents the gene expression level; red means up-regulated genes, and blue means down-regulated genes. (−log2 fold change- > 1 and *p*-value < 0.05).

**Figure 4:**
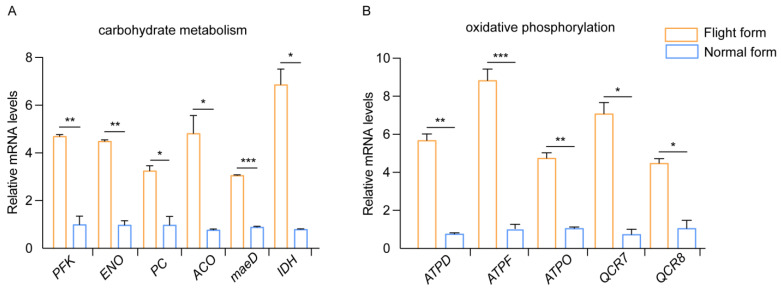
The relative mRNA expression of energy metabolism genes determined by qRT-PCR. (**A**) The expression level of carbohydrate metabolism genes. (**B**) The expression level of oxidative metabolism genes. * *p* < 0.05, ** *p* < 0.01, *** *p* < 0.001 (unpaired *t*-test).

To explore the molecular basis of flight trait differentiation, we conducted a transcriptomic analysis of the two forms. A total of 1336 differentially expressed genes were identified (*p* < 0.05). Among them, 477 genes were up-regulated in the normal form compared to the flight form, while 859 genes were down-regulated (Figures S1 and S2). Energy metabolism is important for flight behavior, so we focused on the differentially expressed genes involved in the energy metabolism pathway. Approximately 65 genes were found to be differentially expressed in the carbohydrate and oxidative metabolism systems. The heat map provides an overview of the expression patterns (Figure 3). In the carbohydrate metabolism pathway, phosphofructokinase (PFK) and enolase (ENO) are the key enzymes for glycolysis. Pyruvate carboxylase (PC), aconitate hydratase (ACO), and malate dehydrogenase (maeD) are involved in the tricarboxylic acid (TCA) cycle. Isocitrate dehydrogenase (IDH) is the rate-limiting enzyme of the TCA cycle. We verified the relative mRNA levels of these genes using qRT-PCR, and the results show that these genes are up-regulated in the flight form compared to the normal form. The expression trends of these genes are consistent with the transcriptomic analysis (Figure 4). Furthermore, in the oxidative metabolism pathway, ATP synthase subunit d (ATPD), ATP synthase subunit f (ATPF), ATP synthase subunit O(ATPO), cytochrome b-c1 complex subunit 7 (QCR7), and cytochrome c reductase subunit 8 (QCR8) are involved in ATP synthesis. We also tested the transcription levels of these genes, and the results showed that their patterns are similar to those of the FPKM values based on sequencing, indicating the reliability of the RNA-seq data (Figure 4).

### 3.4. The Flight Form Maintains Higher Energy Metabolism

**Figure 5:**
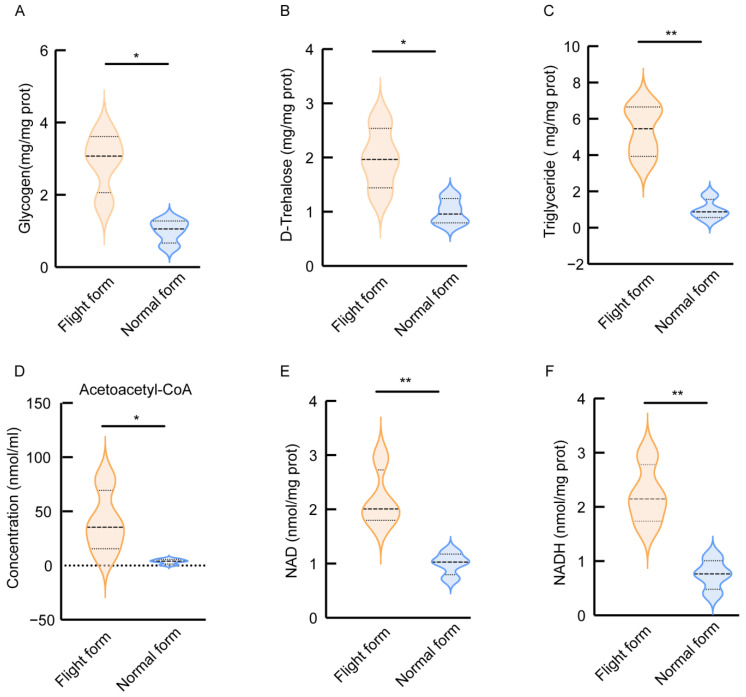
Energy metabolic substance differences between flight form and normal form adult. (**A**–**C**) The differences in relative concentrations of glycogen, D-trehalose, and triglyceride between flight form and normal form adults; (**D**) The absolute concentration of acetoacetyl-CoA. (**E**,**F**) The differences in relative concentrations of NAD and NADH between flight form and normal form adults. * *p* < 0.05, ** *p* < 0.01 (unpaired *t*-test).

At the transcription level, we found an up-regulation of genes involved in the energy metabolism of the flight form compared to the normal form. We determined the differences in the energy metabolism substances between the flight form and the normal form using glycogen, D-trehalose, and triglyceride, which serve as key energy reserves in animal cells [26]. We performed glycogen, D-trehalose, and triglyceride assays to determine the energy metabolism of both forms and found that the flight form had higher relative concentrations of all three [Figure 5A–C]. In addition, we evaluated the concentrations of acetoacetyl-CoA, NAD, and NADH, which are involved in mitochondrial aerobic metabolism. The results show significant differences between the two forms, with a higher absolute concentration of acetoacetyl-CoA in the flight form [Figure 5D]. Similarly, the relative concentrations of NAD and NADH are significantly higher in the flight form compared with the normal form [Figure 5E,F]. These results indicate that the flight form maintains a higher energy metabolism, which enhances its ability to fly.

### 3.5. Abnormal Fecundity of the Flight Form

**Figure 6:**
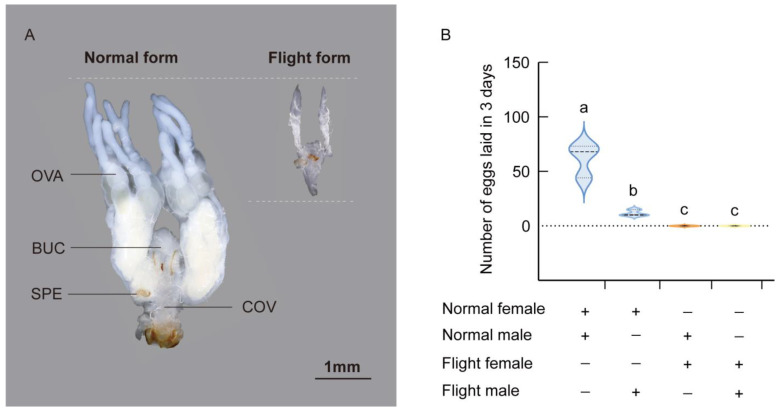
The fecundity difference between flight form and normal form female. (**A**) Photograph of the internal reproductive organs between flight form and normal form female, ovariole (OVA), bursa copulatrix (BUC), spermatheca (SPE), common oviduct (COV); scale bar: 1 mm. (**B**) Number of eggs laid by three females within 72 h of mating. Different letters indicate significant differences among the treatments. (One-way ANOVA and post hoc Tukey’s test, *p* < 0.05).

Expression and metabolism were higher in the flight form at the transcriptional and energy metabolism substance levels. Studies have shown that the pea aphid (*Acyrthosiphon pisum)* experiences a trade-off between reproduction and dispersion [27,28,29]. Therefore, we investigated whether this trade-off was also present in *C. maculatus* and found that the flight form had more flight muscles and greater energy reserves compared to the normal form. Then, we analyzed the fecundity of both forms. There were significant differences in the external morphology of the reproductive organ. The reproductive organ of the normal form was well developed and robust. At the base of the organ, there were a pair of finger-like ovarioles (OVAs), with a triangular bursa copulatrix (BUC) positioned between them. Posteriorly, a thick common oviduct (COV) was located at the end, and a small brownish spermatheca (SPE) was situated near the base of the common oviduct. The reproductive organ of the flight form was only half the length of that of the normal form and appeared significantly shriveled, resulting in a loss of function [Figure 6A]. Furthermore, we investigated the egg-laying behavior of *C. maculatus* by recording the numbers of eggs in different combinations. In 3 days, the normal-form females that mated with normal-form males laid a total of 60 eggs. We also found a decrease in egg deposition when normal-form females mated with flight-form males. Egg-laying was completely blocked in the flight-form females that mated with normal-form males or flight form males [Figure 6B]. Taken together, these data indicate that the mated flight-form females almost lost their fecundity and produced few offspring. This suggests that there is a trade-off between reproduction and dispersion for both forms, with the flight form primarily focusing on dispersion.

## 4. Discussion

Our results reveal that the better development of flight muscles and the robust energy metabolism in the flight form facilitates its ability to fly, leading to adult polyphenism differences in *C. maculatus.* The development of flight muscles and the maintenance of energy metabolism, however, come at a cost to the development of the reproductive organs in the flight forms, resulting in low reproduction rates. The results of our study on *C. maculatus* also support the evidence of a trade-off between dispersion and reproduction.

Our observations of the differences in appearance between the two beetle forms are consistent with findings from a previous study, which suggests that the dimorphism observed in *C. maculatus* might be analogous to the “gregarious” and “solitary” phases observed in *Locusta migratoria* [30]. There are differences in body color between the two forms of *L. migratoria*. Solitary locusts living in low population densities typically exhibit green coloration, while gregarious locusts in larger swarms develop a distinctive pattern of black and brown coloring. The wings of many flightless insects are greatly reduced or even absent. However, the normal form of *C. maculatus* retains its wing structure despite being unable to fly. There are no significant differences in the wing length and width compared to the flight form. It is not yet known whether the subtle differences observed between the anal area of the normal and flight forms are linked to flight ability. The wings of the normal form might serve other functions or may simply be relics that have not yet fully disappeared.

In the same insect species, flightless individuals usually lack some flight muscles compared to those capable of flying (e.g., sexual dimorphism in Lepidoptera by Liu et al.; polyphenism among castes in Isoptera by Zhang et al. and Formicidae by Liu et al.) although there are several different specifications. As Wipfler et al. pointed out, the morphological transformations tied to reduction of flight ability were more dependent on the initial morphology and functional constraints preceding flightlessness [31,32,33]. Thus, it is unsurprising that the normal form lacks the flight-related muscles IIIdlm2, IIIdvm1, 2, and 6 in the metathorax. The flight-related muscles in the mesothorax remain unchanged between two forms. It is possible that the lack of flight ability in the normal form does not affect the opening and closing of the elytra. IIspm7 connects the mesofurca and intersegmental membrane between the meso- and metathorax, likely playing a role in supporting the skeletal structure. IIdvm9, which connects the mesospina with the ventrolateral area of the mesophragma, probably serves a similar function. The lack of flight ability may result in minimal deformation of the thorax in the normal form. The position of the muscle-connecting sclerite is likely influenced by the forces exerted on the thorax. Interestingly, the prothoracic muscles differ between the two forms. With the exception of Idvm10, all other muscles are connected to the cervical sclerites or tentorium, which control neck movements and may be associated with flight ability. Idvm10 connects the profurca and prophragma, which support the skeletal structure and probably also help with thorax deformation.

In insects, carbohydrates and lipids are the most important storage fuels, which are utilized more frequently than other reserves to obtain energy for activity or behaviors [34,35]. Flying animals primarily generate flight energy through the oxidation of carbohydrate and lipids. Honeybees (*Apis mellifera*) and orchid bees (*Euglossa tridentata*) display extremely high metabolic rates during flight [36,37]. In locusts, carbohydrates are also necessary to provide fuel for the initial stage of flight, the transcription levels of genes involved in carbohydrate metabolism were higher in solitary locusts [3]. Lipid oxidation serves as an energy source for flight in the Colorado beetle [38]. Similarly, we found that the flight form and normal form exhibited differential energy metabolism. The flight form showed higher levels of carbohydrates (glycogen and D-trehalose), triglyceride lipids, mitochondrial energetic storage (acetyl-CoA, NAD and NADH), and gene expression related to energy metabolism compared to the normal form. Thus, the high level of energy metabolism in the flight form helps maintains its flight ability. On the other hand, the protein content is not an energy source but rather primarily used for insect development, maintenance, and reproduction [39,40]. In future research, it would be interesting to study the role of proteins in the development or other behaviors of *C. maculatus*. The trade-off between dispersion and reproduction in insects has been well documented, with a negative correlation between the two [41,42]. Numerous studies have shown that long-winged morphs are capable of flight, exhibit lower fecundity, and tend to delay their first reproduction compared to short-winged morphs [43]. The highly atrophied female reproductive organ of the flight form of *C. maculatus* indicates a loss of function. As a result, the flight form has almost lost its ability to reproduce and produces no offspring. The energy used to develop flight-related structures in the flight-capable form can instead be allocated to reproduction in the normal form. The present findings in female *C. maculatus* also support the evidence of a trade-off between dispersion and reproduction.

## 5. Conclusions

This is the first study to analyze the morphology and molecular characteristics of adult polyphenism in *C. maculatus* using morphological, physiological, and behavioral approaches. Our results reveal that the better development of flight muscles and robust energy metabolism in the flight forms facilitates its ability to fly, leading to differences in adult polyphenism in *C. maculatus.* The development of flight muscles and the maintenance of energy metabolism, however, come at a cost to the development of reproductive organs in the flight form, resulting in low reproduction rates. The results of our study on *C. maculatus* also provide supporting evidence of a trade-off between dispersal and reproduction.

## Figures and Tables

**Table 1:** The primers used in this study.

Gene	Forward Primer	Reverse Primer
PFK	CATCCACCATCAGTAACAACG	CAGTATCCGCCCATAACTTCTA
ENO	ACTCAGCAGACCGAAATAGACG	GAAGGCAGGCACAGGTAGAA
FBP	TTTCCCGCAATGACGACT	TGTGGCTCCCTTATGCTTTT
PC	AGACTGAAAGCCGACGAATC	TCTGAAAGGAACCCATAGCC
ACO	AAATGCGTGCGGTCCAT	TCCGGTGAAGTTACGGTTGT
maeB	CGTGTCAGAAGTTTGGGTTG	GTCCGTCACTACGATTGCTTT
IDH3	TTGTGGTTTGGTTGGTGGTG	GCCAATAACATTGCTGTAGGGT
ATPeF0F	GCTACTACGGAAAGGCCGATAC	ACGACTGACAGCTCCAGCGATA
ATPeF0O	AATGGCAGGCCGAACGATA	GCTTGGTTGCAGCAGAATACA
ATPeF0D	TCCTGTAGCTGGAATGGTTGA	TATGCGAGCATTAGACTCTGC
QCR7	AAAAGGCACAAGATGGGTCA	CAACAGCAAGTGGTGGAACA
QCR8	GCGTACAATCTTTCTGGCTTCA	CCAACTTCGTCCACTGTTCTTT
BTF3	TGTCCGTCAACACGATACCC	CCATGACCAGTGATAGCGAAA

## Data Availability

All analyzed data is available in this paper. However, the raw micro-CT data can be made available on request to the corresponding author.

## Supplementary Materials

The following supporting information can be downloaded at: https://www.mdpi.com/article/10.3390/insects16010029/s1, Figure S1:Volcano plot shows differential gene expression pattern between two forms. Figure S2. The heatmap of differential expressed genes between flight form and normal form.
