# Supplementary material for: Divergence in the Morphology and Energy Metabolism of Adult Polyphenism in the Cowpea Beetle Callosobruchus maculatus
Source: Insects. 2024 Dec 30;16(1):29. doi: 10.3390/insects16010029 (PMC11765952; doi:10.3390/insects16010029)
Supplement: Supplementary file 1 [file insects-16-00029-s001.zip › Supplementary Material.pdf]

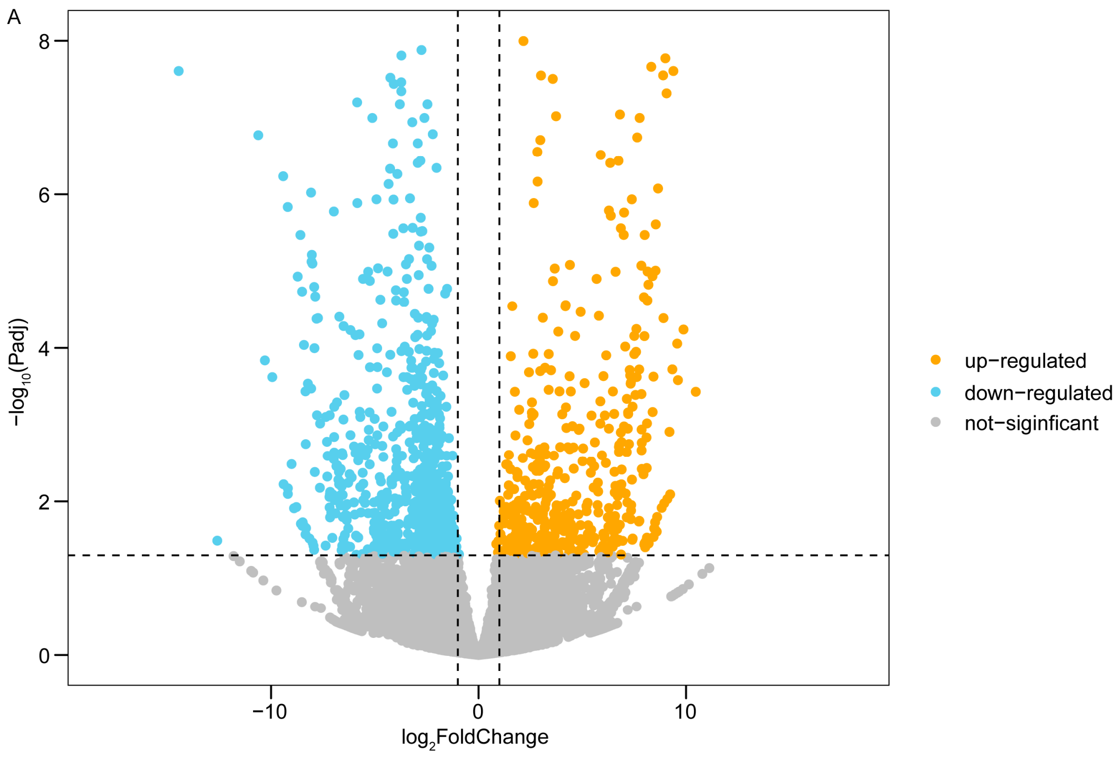

Figure S1. Volcano plot shows differential gene expression pattern between two forms.

Volcano plot based on Fold change (Normal form/ Flight form) and *p*-value. In particular, blue or red dots indicate the presence of significantly down-regulated or up-regulated genes, respectively. Gray dots were not-significant different genes. Volcano plot were acquired by ggplot package.

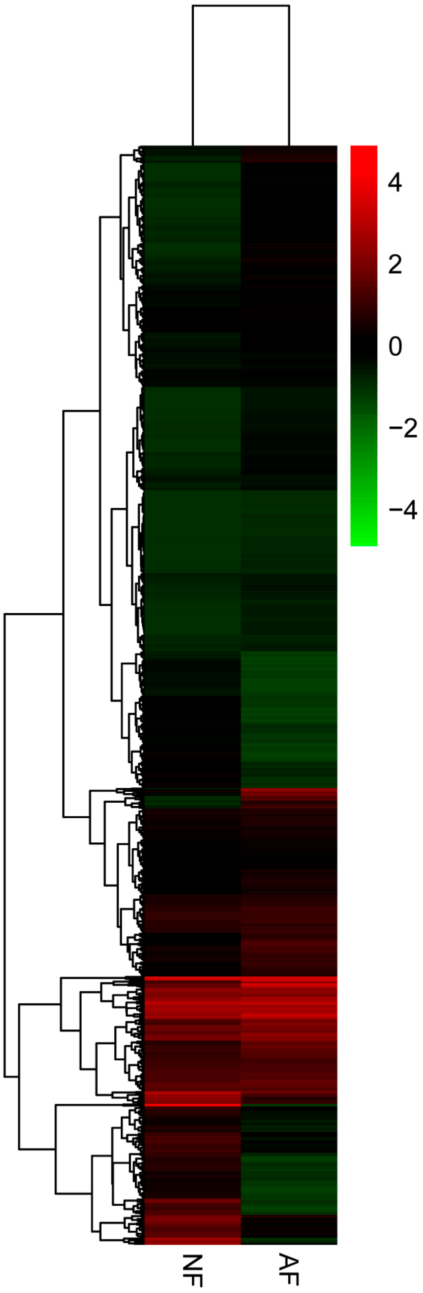

Figure S2. The heatmap of differential expressed genes between flight form and normal form.
