# Supplementary figures and images for: Divergence in the Morphology and Energy Metabolism of Adult Polyphenism in the Cowpea Beetle Callosobruchus maculatus
Source: Insects. 2024 Dec 30;16(1):29. doi: 10.3390/insects16010029 (PMC11765952; doi:10.3390/insects16010029)

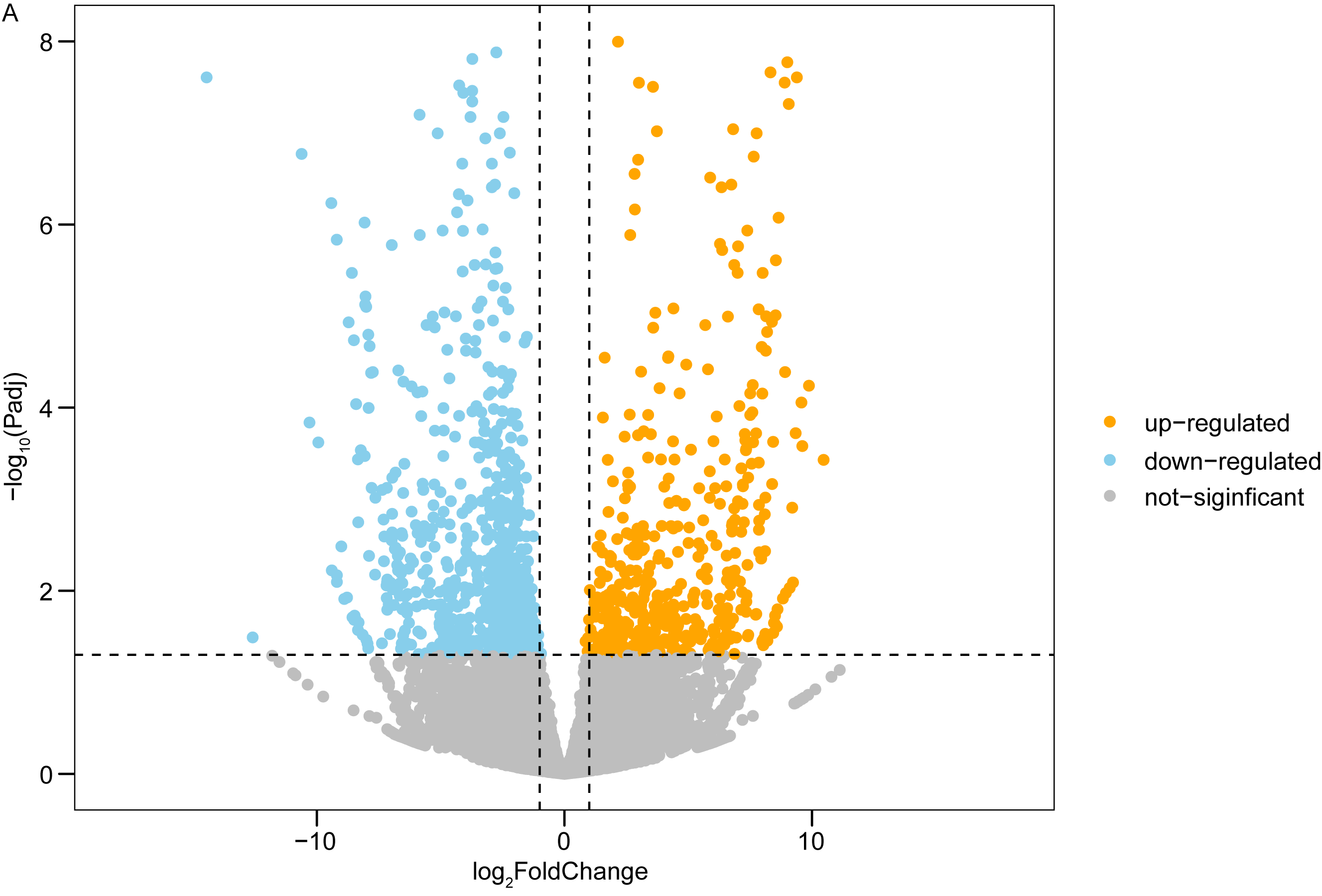

Supplement: Supplementary file 1 [file insects-16-00029-s001.zip › Figure_S1.tif]

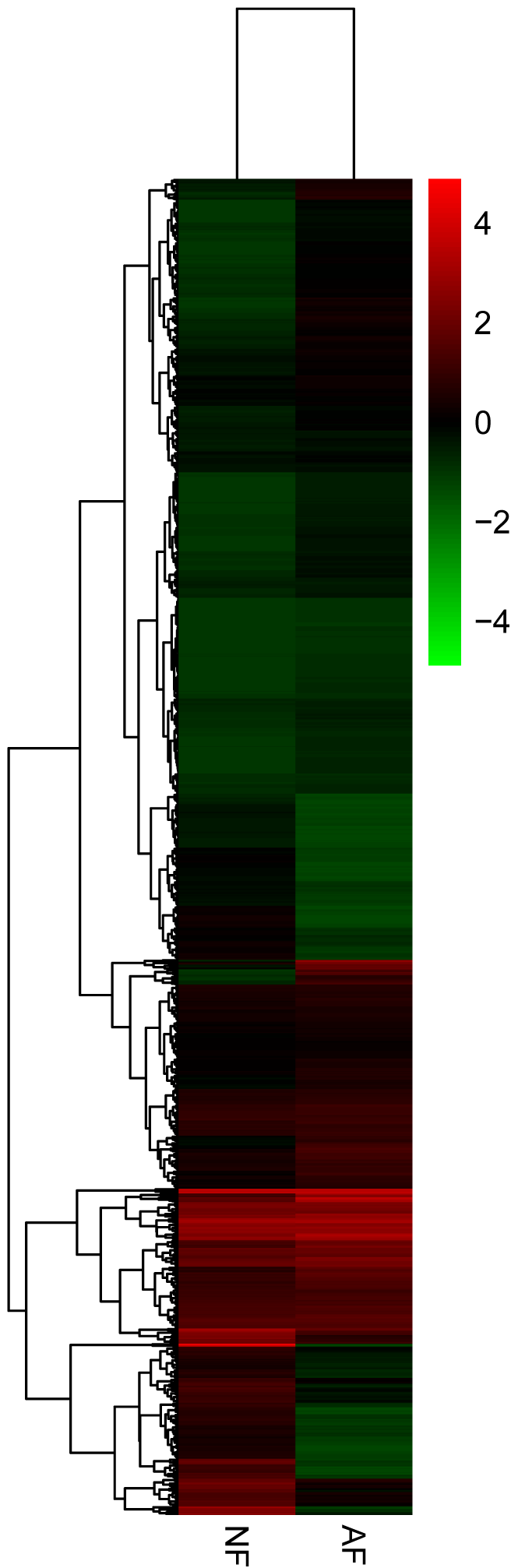

Supplement: Supplementary file 1 [file insects-16-00029-s001.zip › Figure_S2.tif]
